# Detecting cognitive impairment in patients with Parkinson’s disease with a brief cognitive screening tool: the Addenbrooke’s Cognitive Examination (ACE)

**DOI:** 10.1590/S1980-57642009DN20300006

**Published:** 2008

**Authors:** Anabel Chade, María Roca, Teresa Torralva, Ezequiel Gleichgerrcht, Nicolás Fabbro, Gonzalo Gómez Arévalo, Oscar Gershanik, Facundo Manes

**Affiliations:** 1 Institute of Cognitive Neurology (INECO), Buenos Aires, Argentina.; 2 Institute of Neurosciences at Favaloro Foundation, Buenos Aires, Argentina.

**Keywords:** Parkinson’s disease, Alzheimer’s disease, frontotemporal dementia, screening tools, Addenbrooke’s Cognitive Examination (ACE)

## Abstract

Detecting cognitive impairment in patients with Parkinson’s disease is crucial for good clinical practice given the new therapeutic possibilities available. When full neuropsychological evaluations are not available, screening tools capable of detecting cognitive difficulties become crucial.

**Objective:**

The goal of this study was to investigate whether the Spanish version of the Addenbrooke’s Cognitive Examination (ACE) is capable of detecting cognitive difficulties in patients with Parkinson’s disease and discriminating their cognitive profile from patients with dementia.

**Methods:**

77 early dementia patients (53 with Alzheimer’s Disease and 24 with Frontotemporal Dementia), 22 patients with Parkinson’s disease, and 53 healthy controls were evaluated with the ACE.

**Results:**

Parkinson’s disease patients significantly differed from both healthy controls and dementia patients on ACE total score.

**Conclusions:**

This study shows that the Spanish version of the ACE is capable of detecting patients with cognitive impairment in Parkinson’s disease and is able to differentiate them from patients with dementia based on their general cognitive status.

## Abstract (pt)

A detecção de comprometimento cognitivo em pacientes com doença de Parkinson é crucial para uma boa prática clínica devido às novas possibilidades terapêuticas disponíveis. Quando uma avaliação neuropsicológica completa não está disponível, instrumentos de rastreio capazes de detectar dificuldades cognitivas tornam-se cruciais.

**Objetivo:**

Investigar se a versão espanhola do *Addenbrooke's Cognitive Examination* (ACE) é capaz de detectar dificuldades cognitivas em pacientes com doença de Parkinson e discriminar seu perfil cognitivo de pacientes com demência.

**Métodos:**

77 pacientes com demência leve (53 com doença de Alzheimer e 24 com demência frontotemporal), 22 pacientes com doença de Parkinson e 53 controles saudáveis foram avaliados com a ACE.

**Resultados:**

Os pacientes com doença de Parkinson significativamente diferiram de controles saudáveis e pacientes com demência no escore total do ACE.

**Conclusões:**

Este estudo mostra que a versão espanhola do ACE é capaz de detectar pacientes com comprometimento cognitivo na doença de Parkinson e de diferenciá-los de pacientes com demência baseados no seu estado cognitivo geral.

Parkinson's disease (PD) is a progressive and degenerative condition characterized by a loss of dopaminergic neurons in the pars compacta and pars reticulata of the substantia nigra. Since its early description by James Parkinson in 1817,^1^ emphasis had been placed on the motor triad of bradykinesia, rigidity, and tremor. However, over the past decade, research studies have also started to describe the substantial cognitive and behavioral changes occurring in PD. In this vein, numerous publications have contributed to the characterization of the cognitive profile in PD, with research studies revealing specific fronto-subcortical deficits (including difficulties in memory, executive functions, and visuospatial abilities) as well as more generalized impairment of intellect.^2,3^ In practical terms, cognitive dysfunction in PD can be classified as being either domain-specific, for which the severity of the impairment (generally, dysexecutive) does not fall under the established criteria for dementia, or as a being severe enough to comply with dementia criteria.

Despite increasing acknowledgement of cognitive impairments and dementia in PD, no brief, easy-to-administer cognitive test has been proposed for the detection of cognitive deficits with high specificity for PD. With dementia affecting around 40% of patients with PD and the advancement of new pharmacological therapies for cognitive impairment associated with PD,^4^ readily available screening tools are essential for good clinical practice, especially when extensive neuropsychological batteries are not available or assessment time is limited.

Addenbrooke’s Cognitive Examination (ACE)^5^ is an easy-to-administer yet brief screening test that been shown useful for the detection of dementia, and has been previously validated in Spanish by our group. The ACE does not need *ad hoc* material or specific training for its administration, constituting a practical tool readily available to all health professionals regardless of specialty. Previous studies by our research group have demonstrated the utility of the ACE in detecting early dementia^6^ and differentiating these clinical groups from patients with major depression.^7^

While the original English version of the ACE has revealed its capacity for detecting cognitive deficits in atypical parkinsonian syndromes,^8^ this is the first study to investigate its usefulness in detecting cognitive problems in PD. For this reason, the goal of this study was to evaluate whether the Spanish version of the ACE is capable of detecting cognitive difficulties in patients with PD, and to distinguish the cognitive profile of PD patients from that of patients with dementia such as Alzheimer disease (AD) and frontotemporal dementia (FTD).

## Methods

### Participants

A total of 152 participants were included in this study, 77 of which were diagnosed as having dementia – 53 patients with AD and 24 patients with FTD, 22 diagnosed with PD, and 53 healthy controls. AD diagnosis was based on NINCDS-ADRDA^9^ criteria; FTD diagnosis was reached according to the criteria established by Lund and Manchester.^10^ Severity of dementia symptoms was determined using the Clinical Dementia Rating (CDR) scale^11^ and patients were included in this study if their caregivers reported a CDR of 0.5 or 1 (early stage dementia). Diagnosis of PD was carried out by specialized neurologists according to the UK PD Society Brain Bank clinical diagnostic criteria.^12^ Healthy controls were recruited from a larger pool of participants at the Institute of Cognitive Neurology, and had no history of neurological or psychiatric disorder. All participants were assessed by a specialized neuropsychologist or neuropsychiatrist who was blind to patients’ diagnosis.

Statistical analyses were conducted using the SPSS 15.0 statistical package. One-way ANOVA was used to compare demographic variables between the groups with Scheffé post hoc comparisons when relevant. ANCOVA comparisons with age as a covariant factor were conducted on the ACE sub-domain scores and ACE total score. When appropriate, Bonferroni multiple *post hoc* comparisons were calculated.

## Results

Demographic variables are summarized in Table 1. Mean (*SD*) UPDRS score for PD patients was 22.9 (13.1) at the time of cognitive assessment. Significant differences in age (F_3,148_=9.61, *p*<0.001) were observed specifically between PD and controls (*p*<0.001) but not FTD (p=0.87) nor AD (*p*=0.98). Significant differences were observed for years of education (F_3,148_=9.61, *p*=0.011) between AD and controls only (*p*<0.05). However, no differences were found between PD and controls (*p*< 0.01), FTD (*p*=0.98) or AD (*p*=0.91). As expected, significant differences were found on the MMSE^12^ (F_3,148_=11.3, *p*<0.001) between controls and AD patients (*p*<0.01) but not between controls and PD (*p*=0.99).

**Table 1:** Demographic and clinical profile of the groups.

Group	N	Age	Sex (M/F)	Education	MMSE	CDR
PD	22	72.6 (7.17)**	12/10	11.9 (4.71)	28.7 (10.2)	0.26 (0.26)
AD	53	73.4 (7.25)**	23/30	11.1 (3.82)*	24.3 (3.43)*	0.79 (0.25)
FTD	24	70.6 (7.05)	10/14	12.5 (4.46)	26.5 (2.77)	0.79 (0.25)
Control	53	65.3 (9.77)	21/32	13.9 (4.55)	29.2 (1.17)	-

Values are shown as mean (standard deviation). MMSE: Mini-Mental State Exam; CDR: Clinical Dementia Rating Scale. Scheffé's test:

* p<.05;

** p<.01 in comparison with control group.

Performance on the ACE is shown in Table 2. Regarding ACE total score (F_3,148_=45.5, *p*<0.001), PD differed significantly from controls (*p*<0.001) and AD (*p*=0.001), but not from the FTD group (*p*=0.185). The same pattern was observed for the memory (F_3,148_=40.2, p<0.001) and verbal fluency (F_3,148_=39.7, *p*<0.001) sub-domains. When phonological fluency was analyzed based on the ACE scaled score, however, PD differed significantly from controls and both dementia groups (F_3,148_=26.2, *p*<0.001). On both the orientation (F_3,148_=21.0, *p*<0.001) and the visuoconstruction (F_3,148_=22.6, *p*<0.001) sub-domains, significant differences were found between PD and AD, as well as between the AD and controls, and FTD. On the language sub-domain, performance of PD patients did not differ significantly to any of the groups, while the performance of controls did differ significantly to either dementia group.

**Table 2:** Addenbrooke's Cognitive Examination (ACE) cognitive sub-domain and total scores.

	PD	AD	FTD	Control	F_(3,147)_=
Orientation	9.27 (1.35)	7.77 (1.98)*	8.96 (1.3)	9.92 (0.27)	21.0
Attention	6.77 (1.63)	7.12 (1.34)	7.5 (1.06)	7.94 (0.3)	7.81
Memory	25.7 (6.20)	19.58 (6.97)*	22.67 (5.38)	31.19 (3.46)*	40.2
Verbal fluency	9.91 (2.44)	7.27 (2.72)*	7.42 (2.47)*	11.98 (1.6)*	44.3
Language	26.6 (1.76)	25.46 (2.59)	25.42 (3.16)	27.6 (1.47)	9.98
Visuoconstruction	4.14 (1.39)	2.94 (1.51)*	3.88 (1.12)	4.79 (0.53)	22.6
Phonological fluency	5.36 (1.26)	4.17 (1.54)*	3.88 (1.54)*	6.15 (0.99)	26.2
Semantic fluency	4.54 (1.43)	3.1 (1.57)*	3.5 (1.25)	5.85 (1.06)*	39.7
ACE total score	80.6 (17.4)	70.13 (11.87)*	75.79 (8.67)	93.64 (5.23)*	45.5

Values are shown as mean (standard deviation); Bonferroni

* p<.05 in comparison with PD group.

Table 3 shows the number of subjects in the PD and control groups who scored below the cut-off score suggested by the ACE (83 points for research purposes, 88 points for clinical assessment), and the 23-point cut-off score proposed by the MMSE.^13^ The proportion of PD patients who scored below the ACE clinical cut-off score (54.5%) was four times higher than the number of patients who scored below the cut-off score suggested by the MMSE (13.6%)

**Table 3:** Percentage of subjects scoring below cut-off scores suggested for Addenbrooke's Cognitive Examination (ACE) and MMSE.

	Cut-off score		
	ACE < 83	ACE < 88	MMSE < 23
PD group	41.9%	54.5%	13.6%
Control group	3.77%	9.43%	0%

## Discussion

This study shows that the ACE is capable of detecting the presence of cognitive impairment in patients with PD. Indeed, the higher proportion of PD patients with cognitive impairment detected by the ACE over the MMSE reveals the high potential for this brief screening test to identify cognitive deficits in the PD population.

In particular, patients with PD showed more difficulties than controls on the attention, memory, and verbal fluency sub-domains, which is consistent with the hypothesis of fronto-subcortical alterations in this pathology. Our patients did not show difficulties in language or visuoconstruction tasks. Overall, the low total ACE score for PD patients reveals the high sensitivity of this screening test in detecting cognitive impairment.

Moreover, our study shows that the Spanish version of this screening tool is capable of differentiating PD patients from incipient forms of dementia. Patients with PD presented higher overall ACE scores, as well as better performance on tasks of verbal fluency than both AD and FTD groups. In contrast with AD, PD patients showed better temporo-spatial orientation, memory, and visuoconstructive functions. On the other hand, when compared with FTD patients, our PD group differed significantly on the verbal fluency task only, suggesting a profile of frontal dysfunction.

Contrary to the results found by Bak et al.^8^ in atypical parkinsonian syndromes, our study found no differences between phonological and semantic verbal fluency tasks within the PD group. However, it is important to highlight that our study showed that the Spanish version of the ACE is capable of detecting difficulties in the semantic verbal fluency task in PD. This finding is especially important because recent studies^14^ have demonstrated that early problems on semantic fluency tasks, together with difficulties in copying simple figures, are strong predictors of which patients will go on to develop dementia. Thus, the ACE may be a tool able to contribute to the early identification of patients who can benefit from early pharmacological and non-pharmacological strategies aimed at preventing cognitive deterioration.

It is thought that substantia nigra pathology is not sufficient to explain dementia associated with PD, supporting the idea that other subcortical and cortical nuclei are involved in the disorder.^4^ The chemical deficits associated with the cognitive impairments seem to involve a loss of cholinergic, dopaminergic, and noradrenergic innervations. Dementia associated with PD is related to poorer quality of life of both patients and their caregivers, with rapid functional impairment. For this reason, the design of brief, easy-to-administer, and sensitive tools for the detection of cognitive impairment associated with PD will allow early treatment leading to improved quality of life for patients and caregivers alike.

In summary, to the best of our knowledge this is the first study to show that the Spanish version of the ACE is a useful tool for the detection of cognitive impairments associated with PD. In addition, our study demonstrates that the cognitive profile of this patient population can be distinguished from the profile of incipient forms of dementia by using this brief screening tool. The ACE can therefore be used as quick yet efficient tool in studying the cognitive problems associated with PD.

## References

[1] Parkinson J (2002). An Essay on the Shaking Palsy. J Neuropsychiatry Clin Neurosci.

[2] Muslimovic D, Post B, Speelman JD, Schmand B (2005). Cognitive profile of patients with newly diagnosed Parkinson
disease. Neurology.

[3] Foltynie T, Brayne CE, Robbins TW, Barker RA (2004). The cognitive ability of an incident cohort of Parkinson's
patients in the UK. The CamPaIGN study. Brain.

[4] Emre M (2003). Dementia associated with Parkinson's disease. Lancet Neurol.

[5] Mathuranath PS, Nestor PJ, Berrios GE, Rakowicz W, Hodges JR (2000). A brief cognitive test battery to differentiate Alzheimer's
disease and frontotemporal dementia. Neurology.

[6] Sarasola D, Calcagno ML, Sabe L (2005). El Addenbrooke's Cognitive Examination en español para el
diagnóstico de demencia y para la diferenciación entre
enfermedad de Alzheimer y demencia frontotemporal. Rev Neurol.

[7] Roca M, Torralva T, López P, Marengo J, Cetkovich M, Manes F (2008). Diferenciación entre demencias en estadio inicial y
depresión utilizando la versión española del
Addenbrooke's Cognitive Examination. Rev Neurol.

[8] Bak TH, Rogers TT, Crawford LM, Hearn VC, Mathuranath PS, Hodges JR (2005). Cognitive bedside assessment in atypical parkinsonian
syndromes. J Neurol Neurosurg Psychiatry.

[9] McKhann G, Drachman D, Folstein M, Katzman R, Price D, Stadlan EM (1984). Clinical diagnosis of Alzheimer's disease: report of the
NINCDS-ADRDA Work Group under the auspices of department of Health and Human
Services Task Force on Alzheimer's Disease. Neurology.

[10] Neary D, Snowden JS, Gustafson L (1998). Frontotemporal lobar degeneration: a consensus on clinical
diagnostic criteria. Neurology.

[11] Hughes CP, Berg L, Danzinger WL, Coben LA, Martin RL (1982). A new clinical scale for staging of dementia. Br J Psychiatry.

[12] Gibb WR, Lees AJ (1988). The relevance of the Lewy body to the pathogenesis of idiopathic
Parkinson's disease. J Neurol Neurosurg Psychiatry.

[13] Folstein MF, Folstein SE, McHugh PR (1975). "Mini-Mental State": A practical method for grading the cognitive
state of patients for the clinician. J Psychiat Res.

[14] Williams-Gray CH, Foltynie T, Brayne CEG, Robbins TW, Barker RA (2007). Evolution of cognitive dysfunction in an incident Parkinson's
disease cohort. Brain.

